# Chronic alcoholism-mediated metabolic disorders in albino rat testes

**DOI:** 10.2478/intox-2014-0023

**Published:** 2014-12-30

**Authors:** Ganna M. Shayakhmetova, Larysa B. Bondarenko, Anatoliy V. Matvienko, Valentina M. Kovalenko

**Affiliations:** General Toxicology Department, SI “Institute of Pharmacology & Toxicology”, National Academy of Medical Sciences of Ukraine, Kyiv, 03680, Ukraine

**Keywords:** chronic alcoholism, CYP3A2, DNA fragmentation, free amino acids, testes, rats

## Abstract

There is good evidence for impairment of spermatogenesis and reductions in sperm counts and testosterone levels in chronic alcoholics. The mechanisms for these effects have not yet been studied in detail. The consequences of chronic alcohol consumption on the structure and/or metabolism of testis cell macromolecules require to be intensively investigated. The present work reports the effects of chronic alcoholism on contents of free amino acids, levels of cytochrome P450 3A2 (*CYP3A2*) mRNA expression and DNA fragmentation, as well as on contents of different cholesterol fractions and protein thiol groups in rat testes. Wistar albino male rats were divided into two groups: I – control (intact animals), II – chronic alcoholism (15% ethanol self-administration during 150 days). Following 150 days of alcohol consumption, testicular free amino acid content was found to be significantly changed as compared with control. The most profound changes were registered for contents of lysine (–53%) and methionine (+133%). The intensity of DNA fragmentation in alcohol-treated rat testes was considerably increased, on the contrary *CYP3A2* mRNA expression in testis cells was inhibited, testicular contents of total and etherified cholesterol increased by 25% and 45% respectively, and protein SH-groups decreased by 13%. Multidirectional changes of the activities of testicular dehydrogenases were detected. We thus obtained complex assessment of chronic alcoholism effects in male gonads, affecting especially amino acid, protein, ATP and NADPH metabolism. Our results demonstrated profound changes in testes on the level of proteome and genome. We suggest that the revealed metabolic disorders can have negative implication on cellular regulation of spermatogenesis under long-term ethanol exposure.

## Introduction

The present environmental/lifestyle impact on spermatogenesis is an important health issue (Sharpe, 2010). Spermatogenesis is a highly synchronized, regular, continuous and extremely complex process of cellular differentiation, by which a spermatogonial ‘stem cell’ is gradually transformed into a highly specialized haploid spermatozoon (Cheng & Mruc, 2010). The process of spermatogenesis is not initiated until puberty and is then maintained throughout the rest of life in normal men. It is thus only during this period that the spermatogenic process itself is directly vulnerable to adverse effects resulting from the lifestyle of man and/or his exposure to toxic agents from the general environment, or as a result of his occupation. Of the Western lifestyle factors commonly suspected to have adverse effects on health, smoking and alcohol consumption usually come top of the list (Sharpe, 2010). Most studies that included alcohol as a point of investigation have failed to show a significant impact on sperm counts, at least among those with moderate alcohol consumption (Marinelli *et al*., 2004; Martini *et al*., 2004). In contrast, in chronic alcoholics, there is good evidence for impairment of spermatogenesis and reductions in sperm counts and testosterone levels (Villalta *et al*., 1997; Muthusami & Chinnaswamy, 2005). The mechanisms for these effects have not yet been studied in detail (Sharpe, 2010).

Ethanol can cause disturbance in the main metabolic pathways (Zakhari, 2006). Alcohol abuse has a negative effect on all three factors that influence the male reproductive function: hypothalamus-hypophysis-gonad system, endocrine glands, and hormones (Emanuele & Emanuele, 2001). Despite a number of data addressed to the effects of ethanol on the balance of reproductive hormones, changes in the metabolism of proteins, nucleic acids, lipids and carbohydrates in male gonads have not yet been investigated properly. Thus, the effects of chronic alcohol consumption on the structure and/or metabolism of testis cell macromolecules call for intensive studies. In our previous study we showed that 150 days of 15% ethanol self-administration in male rats led to a considerable rise of cytochrome P450 2E1 (CYP2E1) mRNA and protein in testes. Such changes were accompanied by profound spermatogenesis disorders (cauda epididymal sperm quantitative and qualitative parameters deterioration, destructive changes in the spermatogenic epithelium, and decrease of spermatogenic index) (Shayakhmetova *et al*., 2013). Testis CYP2E1 is localized in the Leydig cells, where testosterone biosynthesis takes place (Jiang *et al*., 1998; Forkert *et al*., 2002). In our opinion, the activation of CYP2E1-dependent ethanol-metabolizing systems in steroidogenic cells could determine at least part of the negative effects of alcohol on testes. On the other hand, we suppose that harmful consequences for male reproductive organs mediated by chronic alcohol consumption could be partially a result of degenerative processes caused by complex disturbances in the protein, lipid, and nucleic acid metabolisms. It is particularly the contribution of chronic alcohol intake to changes in the metabolism of testicular amino acids that is not known, especially its influence on any particular amino acid content and function. Similarly does the role of alcohol-mediated individual amino acid changes and their combined effects on testicular proteins and the structure and/or metabolism of nucleic acids remain unclear.

Based on these facts and considering that alcoholism is a chronic disease highly prevalent in the world population, the present work reports the effects of chronic ethanol consumption on free amino acids, levels of cytochrome P450 3A2 (*CYP3A2*) mRNA expression and DNA fragmentation, as well as on contents of different cholesterol fractions and proteins thiol groups in rat testes.

## Material and methods

Wistar albino male rats, initial body weight of 150–170 g, were used in the study. They were kept under controlled temperature (from 22 °C to 24 °C), relative humidity of 40% to 70%, lighting (12 h light-dark cycle), and on a standard pellet feed diet (“Phoenix” Ltd., Ukraine). The study was performed in accordance with the recommendations of the European Convention for the Protection of Vertebrate Animals Used for Experimental and other Scientific Purposes and approved by the Institutional Animal Care and Use Committee. For the experimental (chronic alcoholism) model, reproducing male rats were selected according to the method for measuring voluntary alcohol self-administration in rats, which provides a continuous choice between an alcohol solution and water (two-bottle preference test) (Richter & Campbell, 1940).

Six selected rats were used for chronic alcoholism modeling by replacing water with a 15% ethanol solution during 150 days. The consumption of 15% ethanol was measured as ml and was calculated as g/kg/day of pure ethanol. On this regimen, the daily ethanol consumption was on average 10 g/kg/day. Six intact male rats (of the same age and weight) were used as controls. From the beginning of the experiment, they were kept in the same conditions as the experimental animals, but were given only water *ad libitum*.

After 150 days, both the experimental and control rats were sacrificed under a mild diethyl ether anesthesia by decapitation. The right testis was used for histochemical analysis and the left testis for other investigations.

For enzyme histochemistry, unfixed, 5-µm thick cryostat sections were cut at –18 °C. Succinic dehydrogenase (SDH) activity in the testes was demonstrated by the method of Nachlas *et al*. (Nachlas *et al*., 1957), using nitro blue tetrazolium (Nitro BT) as an electron acceptor. Sections were incubated in the substrate medium containing tetrazolium for 15 min at 37 °C. Appropriate testis sections incubated without substrates were used as controls. Blue diformazan granule deposits were taken as indicative of enzyme activity.

The activity of lactic dehydrogenase (LDH) was histochemically detected according to the technique of Hess *et al*. (Hess *et al*., 1958) as described by Pearse (Pearse, 1968). The sections were incubated in the substrate solution containing Nitro BT and nicotinamide adenine dinucleotide for 15 min at 37 °C. Appropriate testis sections incubated without substrates were used as controls. Violet diformazan deposits were taken as manifestations of LDH activity.

To testis homogenate samples (1 ml) equal volumes of 3% sulfa-salicylic acid were added and the obtained mixtures were left for 10 minutes in the refrigerator at 4 °C. The formed sediments were removed by centrifugation (5,000 *g*, 10 min, 4 °C) (Kuchmerovska *et al*., 2008). Supernatants contained free amino acids from testes. Contents of free amino acids were determined on the amino acid analyzer AAA-881 (Czech Republic).

The total cholesterol content in testis homogenates was investigated according to a standard spectrophotometric method (Kates, 1972).

The contents of protein SH-groups in testis homogenates were determined with Ellman's reagent (Sedlak & Lindsay, 1968).

The total protein contents in testis homogenates were assessed by the method of Lowry *et al*. (Lowry *et al*., 1951).

The DNA from the testes was isolated by a modified method from Current Protocols in Toxicology (Zhivotosky & Orrenius, 2001). The tissue was homogenized and digested in digestion buffer (100 mM NaCl; 10 mM Tris-HCl; 25 mM EDTA, pH 8; 0.5% SDS and freshly added 0.1 mg/ml proteinase K) (Sigma-Aldrich, Inc., USA)) (1:1.2 mg/ml) with shaking at 50 °C for 15 h. RNA was degraded by incubation of the samples with 1–100 mg/ml thermostable RNAse H for 1.5 h at 37 °C. DNA was extracted with an equal volume of phenol:chloroform:isoamyl alcohol (25:24:1) and centrifuged for 10 min at 1,700 *g*. Then the DNA was precipitated by adding 0.5 vol 7.5 M ammonium acetate and 2 vol 100% ethanol to the aqueous layer; samples were separated by centrifugation at 1,700 *g* for 5 min, rinsed with 70% ethanol, and air-dried. Pellets were dissolved in TBE buffer (10 mM Tris-HCl and 1 mM EDTA, pH 8) and then fractionated through 2% agarose gels (50–60 V; 3.5 h). After electrophoresis, the gels were stained with ethidium bromide and visualized under a UV transilluminator (BIORAD, USA). Analysis of electrophoresis data was carried out with Quantity One Software (USA).

The expression *CYP3A2* (ortholog of human *CYP3A4* (Jäger *et al*., 1999)) mRNA in testes was determined by a reversed transcriptase polymerase chain reaction (RT-PCR). Testis samples (50 mg) were collected, quickly frozen in liquid nitrogen, and stored at –80 °C before RNA extraction. The isolation of total mRNA was carried out with a TRI-Reagent (Sigma, USA). The integrity and concentration of RNA was analyzed in a 2% agarose gel. First-strand complementary DNA (cDNA) was synthesized using a First-Strand cDNA Synthesis Kit (Fermentas, Germany). The reaction mixture contents for PCR, amplification protocol, and specific primers for the CYP2E1 gene were chosen according to Jäger *et al*. (1999). The primer sequences: sense, 5’-TACTACAAGGGCTTAGGGAG-3’ and anti-sense, 5’-CTTGCCTGTCTCCGCCTCTT-3’. RT-PCR with primers of house-keeping gene β-actin sense, 5’-GCTCGTCGTCGACAACGGCTC-3’ and antisense 5’-CAAACATGATCTGGGTCATCTTCT-3’) were carried out for internal control. All of the primers were synthesized by “Metabion” (Germany). The MyCycler thermocycler (BioRaD, USA) was used for amplification. PCR products (CYP3A2 – 349 bp and β-actin – 353 bp) were separated in a 2% agarose gel, stained with ethidium bromide, and visualized under a UV transilluminator (BIORAD, USA). Data analysis was carried out with Quantity One Software (USA) and presented in relative units as the ratio of *CYP3A2* mRNA and β-actin mRNA contents.

The obtained data were calculated and expressed as the mean ± standard error of the mean (mean±S.E.M.). Data were compared using Student's t-test. Differences were considered to be statistically significant at *p*<0.05.

## Results

Investigation of chronic alcoholism effects on rat testis pools of free amino acids (Table 1) showed that statistically significant changes, as compared with control, were registered for 5 amino acids and for the total sum. Contents of lysine, arginine, serine, glycine were decreased while contents of methionine were increased. The contents of lysine (–53%) and methionine (+133%) exhibited the most profound changes.

**Table 1:** Rat testes contents of free amino acids (mg/ 100 g of moist tissue) after 150 days of 15 % ethanol consumption.

Amino acid	Animal groups	
	Control	Experimental
Lysine	2.60±0.30	1.20±0.10*
Histidine	0.40±0.10	0.36±0.12
Arginine	1.30±0.20	0.63±0.07*
Ornithine	0.60±0.20	0.52±0.10
Aspartic acid	9.70±3.10	8.80±2.50
Threonine	2.20±0.10	2.40±0.10
Serine	2.60±0.10	1.80±0.09*
Glutamic acid	34.60±3.50	33.20±4.10
Proline	3.00±0.80	3.90±0.40
Glycine	11.80±2.30	6.40±0.70*
Alanine	8.00±1.30	9.10±1.00
Cysteine	0.70±0.20	0.50±0.10
Valine	0.80±0.20	0.36±0.08
Methionine	0.30±0.10	0.70±0.10*
Isoleucine	0.60±0.20	0.34±0.05
Leucine	0.70±0.20	0.44±0.09
Tyrosine	1.10±0.,30	0.62±0.04
Phenylalanine	1.40±0.40	1.98±0.06
Glutamine	16.10±3.50	12.70±2.80
Total sum	108.10±4.70	85.95±2.80*

mean±S.E.M., n = 6

* *p <*0.05 statistically significant in comparison with control

**Figure 1:**
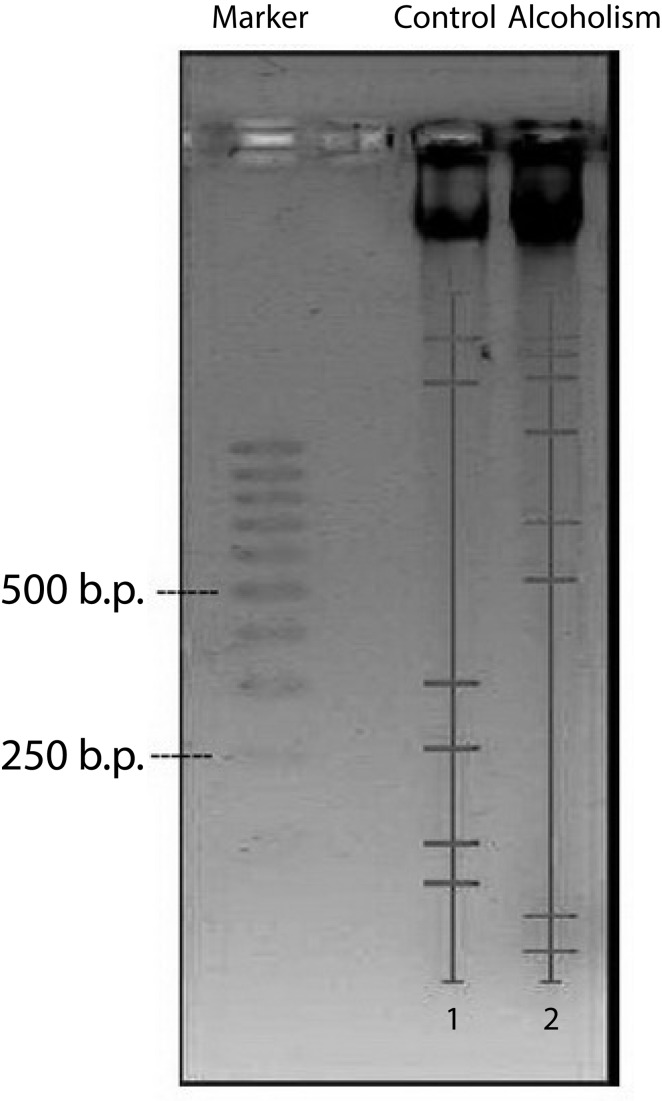
Levels of DNA fragmentation in rat testes. Analysis was carried out using the Quantity One Software

Investigation of rat testes DNA fragmentation demonstrated its essential intensification following 150 days of ethanol administration in comparison with control (Figure 1). In the control group, 6 fractions of DNA fragments with weights 1300, 1100, 300, 250, 100 and 60 b.p. were present. Main fractions of high-weighted DNA fragments had weights of 1300 b.p. and of low-weighted DNA fragments 60 b.p.

In testes of alcohol-treated rats 8 fractions of DNA fragments with weights over 1000 (4 different fractions), 700, 550, 30 and 20 b.p. were present. Main of high-weighted DNA fragments were fractions with weights over 1000 b.p., while among low-weighted DNA fragments fractions were approximately equal (by peak intensity).

**Figure 2:**
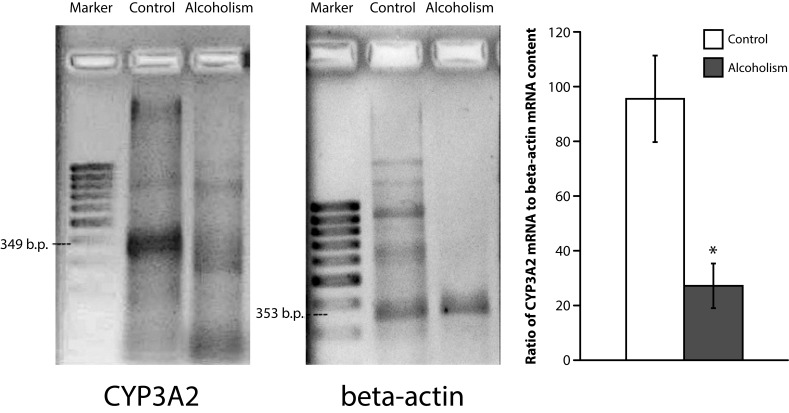
CYP3A2 mRNA in rat testes:**a** – electrophoregram of CYP3A2 and reference-gene β-actin RT-PCR products (arrows indicate appropriate DNA fragments); **b** – average rate of CYP3A2 mRNA expression in rat testes. * *p <*0.05 statistically significant in comparison with control.

A considerable inhibition of *CYP3A2* mRNA expression was indicated in testes of rats with chronic alcoholism (Figure 2). This parameter decreased 3.5 times as compared with control.

At the same time in testes of alcohol-addicted animals the contents of total cholesterol increased by 25%, etherified cholesterol increased by 45%, and protein SH-groups decreased by 13% in comparison with control (Table 2).

**Table 2:** Rat testes contents of total cholesterol and SH-group proteins after 150 days of 15 % ethanol consumption.

Indices	Animals groups	
	Control	Alcoholism
Total cholesterol, µmoles/mg of protein	3.98±0.63	4.98±0.39*
Free cholesterol, µmoles/mg of protein	1.34±0.07	1.51±0.17
Etherified cholesterol, µmoles/mg of protein	2.64±0.68	3.84±0.36*
Protein SH-groups, nmoles/mg of protein	133.06±1,83	115.41±3,92**

mean±S.E.M., n = 6;

* *p <*0.05 statistically significant in comparison with control;

** *p <*0.01 statistically significant in comparison with control

**Figure 3:**
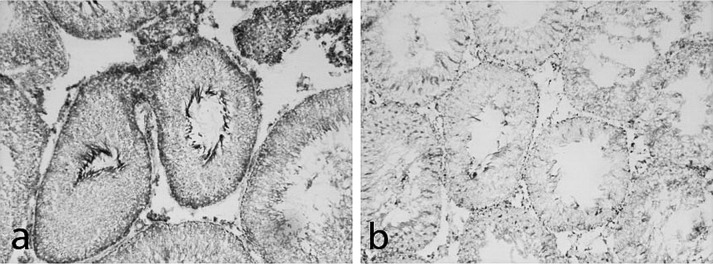
SDH activity in rat testes: **a** – control; **b** – nidal decrease of enzymatic activity following 150 days of 15% ethanol consumption; method of Nachlas *et al*. (1957), 200×.

Evaluation of SDH histo-topography showed a significant decrease of its enzymatic activity in alcoholic rat testes as compared with control (Figure 3). Deposits of blue diformazan granules were focally lower, mainly small granules of diformazan were seen, and cellular cytoplasm was stained pink. Additionally, single polymorphic granules of diformazan were fixed in some seminiferous tubules.

**Figure 4:**
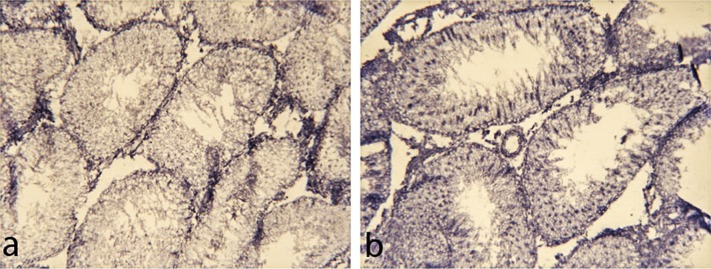
LDH activity in rat testes: **a** – control; **b** – increase of enzymatic activity following 150 days of 15% ethanol consumption; method of Hess *et al*. (1958), 200×.

On the contrary, the activity of LDH increased in all layers of spermatogenic epithelium of alcohol-treated rat testes (Figure 4).

## Discussion

It is particularly the character of amino acid changes provoked by excessive ethanol consumption that points to considerable general disturbances in the metabolism of proteins (Murakami *et al*., 2013). Our results are in good accordance with other authors’ data (Reilly *et al*., 1997) who demonstrated the ability of ethanol to impair protein synthesis.

We detected changes of testis free amino acid contents that can also be taken as evidence of disturbances in metabolism of adenosine triphosphate (ATP) and reduced form of nicotinamide adenine dinucleotide phosphate (NADPH), both at the stage of glycolysis (changes of glycine and serine) and in the citric acid cycle (changes of methionine) (Reilly *et al*., 1997). Our SDH activity data also indicate possible changes in the citric acid cycle. Our assumption on the possibility of ATP and NADPH metabolism violations in testes of alcohol-addicted rats are in good correspondence with our results on testicular LDH activity changes.

Serine and arginine can act as an NO depot, which is not only responsible for blood vessel relaxation but also reacts with the iron atoms (in heme and free state), with superoxide anions, oxygen molecules, hydroperoxide, organic peroxides and peroxide radicals (Stepuro, 2001). Thus, changes of serine and arginine contents in our experiments could adversely affect at the level of NO, peroxidation rates and functioning of the vascular system both in testes and the whole organism (Zhang *et al*., 2005). Arginine could act as an antioxidant and powerful immunomodulator (Pavlov, 1998), as a precursor in biosynthesis of polyamines, which regulate cell proliferation processes in the organism.

Changes of methionine in testicular pools deserve special attention. This amino acid can act as antioxidant and immunomodulator (Pavlov, 1998; Hipkiss, 2012). Methionine is a precursor of taurine – antioxidant and membrane stabilizer. It can influence the composition of tissue lipidome and membrane lipids (Jové *et al*., 2013). Alterations in the methionine cycle are closely connected with cholesterol metabolism violations (also detected in our experiments) and disturbances preceding molecular signs of inflammation (Thomas, *et al*., 2013). This amino acid is also a precursor of polyamines – stimulators and regulators of proliferation processes (Pavlov, 1998; Jung *et al*., 2013). The up-regulation of methionine content in our experiment may be regarded as a defensive mechanism (Jung *et al*., 2013).

It is reasonable to propose that biosynthesis of S-adenosylhomocysteine and homocysteine could be also broken due to excessive alcohol intake. Changes in contents of methionine, glycine and serine – amino acids involved in the synthesis of these compounds (Jung *et al*., 2013; Marks, 1994), point to such a possibility. This supposition is in good accordance with other authors′ data demonstrating that alcohol-induced organ injury is associated with a decreased S-adenosyl-l-methionine/S-adenosyl-l-homocysteine ratio and depletion of mitochondrial glutathione, which has been shown to sensitize cells to tumor necrosis factor (TNF) (Fernández *et al*., 2009). Accumulation of hydroperoxides and disturbances of DNA reparation processes could be a result of changes in homocysteine metabolism (Jung *et al*., 2013; Kovalenko *et al*., 2007).

Changes of such amino acids as glycine in the testis pool are of special importance because of the involvement (along with other amino acids) in glutathione biosynthesis (Marks, 1994; Dimitrova *et al*., 2002). On the other hand, as glutathione realizes trans-membrane transport of amino acids (Marks, 1994; Roth *et al*., 2002; Rennie *et al*., 1998), changes in its contents can cause changes in free amino acid contents (Marks, 1994; Roth *et al*., 2002; Rennie *et al*., 1998).

In the group with chronic alcoholism, we established statistically significant changes in testicular contents of amino acids involved in the biosynthesis of purines and pyrimidines (glycine and serine) (Marks, 1994). Such changes could influence processes of nucleotides, the metabolism of nucleic acids, and the state of cell chromosomes.

DNA is an important molecular target for toxicants (Kovalenko *et al*., 2007) inducing endonucleases for its lethal splitting. Such compounds could inhibit processes of DNA repair by nuclear DNA-polymerases. The level and character of DNA fragmentation are markers of apoptotic processes in the organism (Wang *et al*., 2005). Our results on DNA fragmentation rates in testes of ethanol-treated rats are in good correspondence with other authors′ data (Miñana *et al*., 2002). It should be noted that in adulthood, apoptosis plays a significant role in regulating germ cell development. For example, apoptosis is used as a mechanism for removing damaged germ cells from seminiferous tubules so that they do not continue to differentiate into spermatozoa. Selective deletion of damaged germ cells is clearly a critical component of the mechanisms used to safeguard the genome of a given species. The range of stimuli that will trigger this activity is impressively broad, including various forms of electromagnetic radiation, environmental toxicants, heavy metals and chemotherapeutic agents (Aitken & Baker, 2013).

Ethanol induces apoptosis via 2 different pathways: mitochondrial permeability transition and up-regulation of the expression of CD95-Fas ligand. The overproduction of reactive oxygen species (ROS) by mitochondria, driven by acetaldehyde metabolism, is a common trigger of both mechanisms (Miñana *et al*., 2002). In the organism of alcoholics, apoptosis is caused by increased ROS due to increased availability of the reduced form of nicotinamide adenine dinucleotide (NADH) owing to mitochondrial acetaldehyde metabolism, and it is prevented by blocking the opening of mitochondrial permeability transition pores with cyclosporine A (Miñana *et al*., 2002).

An ability of ethanol to disturb spermatogenesis was recorded in numbers of studies (Villalta *et al*., 1997; Muthusami & Chinnaswamy, 2005; Emanuele & Emanuele, 2001; Martinez *et al*., 2000; El-Sokkary, 2001). On the other hand, when spermatogenesis is disrupted in any way, the germ cells tend to default to an apoptotic state (Aitken & Baker, 2013). Apoptosis during spermatogenesis has also been suggested to play a role in the etiology of spontaneous male infertility in light of the excessively high numbers of apoptotic germ cells observed in testes of some infertile males (Aitken & Baker, 2013).

The testis contains two distinct sets of cytochrome P450 enzymes. One set consists of steroidogenic P450 enzymes (e.g., CYP11A1 and CYP17A1), which are involved in testosterone biosynthesis (Shan *et al*., 1993). The other set comprises those P450 enzymes which play a major role in the biotransformation of hydrophobic xenobiotic compounds to more water-soluble metabolites (Shayakhmetova & Bondarenko, 2013). The physiological role of xenobiotic-metabolizing P450 enzymes in the testis is not definitively known, but it has been proposed that they could catalyze the oxidative biotransformation of lipophilic xenobiotic or endogenous compounds within the testis, which could present important implications for testicular toxicity (Shayakhmetova & Bondarenko, 2013; Schuppe *et al*., 2000). In our previous study, we showed *CYP2E1* mRNA expression and protein content elevation in alcohol-treated rat testes with simultaneous spermatogenesis violations (Shayakhmetova *et al*., 2013). In the present investigations, we studied the effects of long-term ethanol consumption on *CYP3A2* expression in rat testes. CYP3A2 is the rat ortholog of the human enzyme CYP3A4 (Jäger *et al*., 1999). Our results indicated that chronic alcohol exposure significantly down-regulated the testicular *CYP3A2* mRNA level.

Ethanol has been reported to be either an inducer or an inhibitor of CYP3A expression. CYP3A exposure induced P450 3A in primary cultures of human and rat hepatocytes (Kostrubsky *et al*., 1995; DiPetrillo *et al*., 2002). In the CYP3A4-expressing HepG2 cell line, incubation with ethanol increased *CYP3A4* mRNA level and CYP3A activity in a dose-dependent manner (Feierman *et al*., 2003). Several *in vivo* studies indicated a relationship between CYP3A and the duration of ethanol exposure. In rats fed ethanol with the Lieber-DeCarli diet for 7–14 days, both ERND catalytic activities and immunoreactive CYP3A were increased (Roberts *et al*., 1995). In addition, ethanol significantly increased fentanyl *N*-dealkylatase activities in rats fed ethanol for 21 days (Feierman *et al*., 2003). However, in rats fed ethanol diet for 38 days, there was a significant decrease in hepatic testosterone 6-hydroxylase activities (Badger *et al*., 1993). Similarly, Rowlands *et al*. found that CYP3A apoprotein level and testosterone 6-hydroxylase activities decreased in rats fed ethanol diet for 42–55 days (Rowlands *et al*., 2000).

It is known that hepatic *CYP3A* expression is highly regulated by pregnane X receptor (PXR), a member of the nuclear receptor superfamily, regulating gene transcription in a ligand-dependent manner (Kliewer *et al*., 1998; Lehmann *et al*., 1998). However, the results of a previous report (Zhang *et al*., 1999) showed that PXR was not expressed, while *CYP3A2* was demonstrated to be expressed in rat testes (Kim *et al*., 2003). In our opinion, the down-regulation of *CYP3A2* mRNA in the testes by ethanol could indicate its ability to affect *CYP3A2* at the transcription level independently of PXR.

Findings in rodent models have shown that di-2-ethylhexyl phthalate is able to induce *CYP3A* in testes and liver, resulting in intensification of testosterone metabolism (16alpha- and 6beta-hydroxylation increase) (Kim *et al*., 2003). The results of the present study suggest that inhibition of testicular *CYP3A2* mRNA expression could, at least partially, mediate the ability of ethanol to disturb testosterone metabolism and act as an endocrine disruptor.

Our results on cholesterol content changes are in good accordance with other authors’ data demonstrating that chronic ethanol exposure causes significant increase in levels of testicular cholesterol, free fatty acid, phospholipids and triglycerides (Radhakrishnakartha *et al*., 2013). Yet in our experiments we showed for the first time different degrees of changes in different cholesterol fractions.

The Leydig cells of the testis have the capacity to biosynthesize testosterone from cholesterol. Testosterone and its metabolically activated product dihydrotestosterone are critical for the development of the male reproductive system and spermatogenesis (Ye *et al*., 2011). Antiandrogenic chemicals could suppress androgen production in Leydig cells, reduce their number, or bind to the androgen receptors so as to block activation by androgens (Ye *et al*., 2011). We found, along with other authors (Radhakrishnakartha *et al*., 2013), that the high testicular level of cholesterol at the background of the well known ethanol-mediated testosterone synthesis suppression (Orpana *et al*., 1990; Adams *et al*., 1991; Frias *et al*., 2000) may manifest due to direct ethanol interaction with the testosterone biosynthetic pathway and/or metabolic activation pathway.

Our results on the decrease of protein SH-groups in testes of alcohol-treated rats are in good accordance with data of clinical studies on the decrease of total thiol in alcohol abusers (Prakash *et al*., 2008). The alcohol induced formation of free radicals and the oxidative damage in alcoholism have been documented by several authors by measuring various oxidants and antioxidants in the organism (Albano, 2006; Lieber, 1997). Protein thiols represent a prominent biological target for ROS, and their levels can be used as markers of oxidative modification of proteins (Mimić-Oka *et al*., 2001). It has been estimated that proteins can scavenge the majority (50%–75%) of generated reactive species (Davies *et al*., 1991) and much of this function is attributed to the thiol groups present on them. One or more reduced thiol (-SH) groups are essential for the function of many proteins (Topçuoglu *et al*., 2009). Consequences of the damage of protein SH-groups may be impaired enzymatic activity and modified membrane and cellular function, depending on the nature of the vulnerable protein component and the attacking radical species.

Our results suggest that the self-administration of 15% alcohol in rats during 150 days led to multidirectional changes of the activity of testicular dehydrogenases, which play a crucial role in supplying energy needed for various metabolic functions in germ cells (Mathur, 2012). Dehydrogenases form enzyme groups of mitochondrial and cytoplasmic origin, which facilitate many oxidoreduction reactions responsible for generating ATP. LDH and SDH are important oxidoreductases linked to the events of spermatogenesis and androgenesis (Mathur, 2012). LDH activity is mainly of tubular localization, but SDH showed also interstitial activity (Blackshaw & Massey, 1978).

Forming part of complex II of the respiratory chain, SDH, is situated at the intersection of the citric acid cycle and oxidative phosphorylation. This combination of functions places SDH at the center of two essential energy-producing metabolic processes of the cell (Cervera *et al*., 2009). SDH enzymatic activity is associated with maturation of germ cells (Hodgen & Sherins, 1973). The decrease of SDH activity in testes of experimental animals could be evidence of metabolic alterations in spermatogenic epithelium and reduction of the energetic resources of germ cells following prolonged ethanol administration.

It is well known that spermatogonia may utilize glucose as the major energy substrate, but spermatocytes and spermatids suffer a rapid decline in their ATP content in glucose-supplemented media and require lactate/pyruvate for the maintenance of their ATP concentrations (Jutte *et al*., 1981).

Glucose transport into the cell and the lactate LDH isoenzyme system, which reversibly catalyzes the inter-conversion of pyruvate and lactate, are biochemical steps which participate in the regulation of lactate production (Riera *et al*., 2002). Facilitated Sertoli cell glucose transport across the plasma membrane is mediated by the carrier protein termed glucose transporter 1, the only glucose transporter so far demonstrated in this cell. As for the LDH isoenzyme system, increments in lactate production in Sertoli cells have been correlated with an increase in the LDH5 isoenzyme containing four subunits A. Glucose transport through the plasma membrane and LDH A mRNA levels are regulated in a distinct manner by FSH, interleukin 1, TNF and epidermal growth factor – factors that modify the lactate production of Sertoli cells (Riera *et al*., 2002).

Chemically induced stress causes elevated LDH activity, which can be used as a good diagnostic tool in toxicology (Ksheerasagar & Kaliwal, 2013). In our experiment the elevation of LDH activity in testes of alcohol-treated rats indicates enhancement in the extent of lactate and pyruvate mobilization into the citric acid cycle, and it could reflect the compensatory capability of gonads.

## Conclusions

Thus investigation of chronic alcoholism effects on testicular levels of free amino acids, rates of *CYP3A2* mRNA expression and DNA fragmentation processes, as well as changes in cholesterol and protein thiol group contents allowed us to obtain complex estimation of this pathologic influence in male gonads, especially on the metabolism of amino acids, proteins, ATP and NADPH. Our results demonstrated profound changes in testes on the level of proteome and genome. We suggest that the revealed testicular metabolic disorders could have negative implications on cellular regulation of spermatogenesis under long-term ethanol exposure.
